# ‘Alright my lovely’: The use of terms of endearment as a mitigation device in the care of people living with dementia in the acute hospital environment

**DOI:** 10.1177/13634593241238856

**Published:** 2024-04-04

**Authors:** Lauren Bridgstock, Alison Pilnick, Sarah Goldberg, Rowan H Harwood

**Affiliations:** University of Nottingham, UK; Manchester Metropolitan University, UK; University of Nottingham, UK; University of Nottingham, UK; Nottingham University Hospitals NHS Trust, UK

**Keywords:** ageing and lifecourse, conversation analysis, health, health policy, patient-physician relationship, quality of life

## Abstract

This paper examines how terms of endearment (ToE) are used as a mitigation device in interactions between staff and people living with dementia (PLWD) in the acute hospital environment. ToE are often discouraged in training for healthcare staff. However, this research demonstrates that they are still commonly used in practice. Using conversation analysis, video and audio data were examined to identify the interactional functions of ToE. Analysis showed that ToE play an important role in mitigating potentially face-threatening actions such as when patients are asked to repeat hard-to-interpret talk, or when patient agency is compromised through instruction sequences or having necessary healthcare tasks undertaken. The success of this mitigation is sensitive to the specific interactional circumstances, as well as the responsiveness of the HCP to the patient’s voiced concerns. These findings have implications for healthcare practice, training and wider care of PLWD.

## Introduction

An estimated 57 million people have dementia worldwide, and this is expected to rise to 153 million by 2050 (GBD 2019 Dementia Forecasting Collaborators, 2022). Apart from affecting memory, dementia can involve many other symptoms such as delusions, hallucinations, aggression, depression, anxiety, apathy, disinhibition, motor disturbances, problems with appetite and eating and agitation (including resistance to care) (Kales et al., 2015). Many people living with dementia (PLWD) will be admitted to hospital, often in an emergency (Goldberg et al., 2012; National Institute for Health and Care Excellence (NICE), 2023). Approximately one quarter of all hospital beds in the UK are occupied by PLWD (NICE, 2023; Sampson et al., 2009), with numbers increasing in recent years (Alzheimer’s Society, 2020; NICE, 2023; Torjesen, 2020). PLWD often also experience delays in leaving hospital and longer stays (NICE, 2023). Research has repeatedly highlighted negative outcomes associated with hospitalisation of PLWD, including increased likelihood of falls, disorientation, functional decline, malnutrition and death (e.g. Dewing and Dijk, 2016; Featherstone and Northcott, 2020; Røsvik and Rokstad, 2020; Sampson et al., 2009). There is a need to consider how the care experiences of PLWD can be better understood and improved.

Communication is at the heart of healthcare delivery (Drew et al., 2001), but this is recognised as challenging in situations involving PLWD, and staff report feeling under-trained in managing it (Griffiths et al., 2014). This has wider implications, such as the views of PLWD potentially being neglected in medical discussions (Graham, 2004) or necessary tasks being rejected or resisted. Communication and inclusion of PLWD in interaction has been successfully analysed in other care contexts (e.g. see Hydén et al., 2023; Slocombe et al., 2024). A key part of communication in hospital is how patients are addressed by healthcare professionals, as there is a tension between causing potential offence if too familiar or inattentive to status and demonstrating solidarity and a caring disposition (Bowie, 1996; Wood and Ryan, 1991).

One widely contested area is the use of terms of endearment (ToE). These are routinely discouraged in training for nursing home staff (e.g. Williams et al., 2016) and healthcare professionals (HCPs) such as nurses (Laskowski-Jones, 2015). Furthermore, the National Service Framework for Older People (Department of Health, 2001) instructs that staff should use an older person’s preferred form of address and relate to them as a competent adult. NICE guidelines give similar instructions (NICE, 2012). However, as this paper will show, ToE are commonly used in practice by a range of experienced and skilled HCPs. This implies that these terms may fulfil a function, which will be examined below.

There is no definitive definition of the phrase ‘terms of endearment’. In academic literature, researchers often simply use the phrase and provide some examples such as ‘*honey*’ or ‘*sweetheart*’, (Brown and Draper, 2003: 16) or ‘*Honey*’, ‘*Sweetie’, ‘Grandma’, ‘Babe’* and *‘Sunshine*’. (Williams et al., 2017a: 9). Khalil and Larina suggest ‘*Terms of endearment can be regarded as expressions that convey intimacy; they are usually used to address those who are close to the speaker*’ (2022: 29). This focus on the addressee is important, particularly if the receiver is an older individual. Shaw and Gordon (2021: 6) categorise ToE as a form of elderspeak (a type of communication used towards older adults) and specify that in this circumstance their use is ‘*inappropriate of the interlocutor relationship*’. Likewise, Williams et al. (2003: 246) note that their use is ‘*inappropriately intimate’.* A limitation with these definitions is the conflation of different types of ToE. It could be argued for instance that ‘Grandma’, implies a relationship that is more familiar (and potentially familial) than something like ‘Sweetie’. Furthermore, some of these terms are gendered, and some are not, which may also influence reception.

This categorisation of ToE as a form of inappropriate or infantilising communication appears to be commonly accepted (see Schnabel et al., 2021; Shaw et al., 2022; Williams et al., 2016, 2017a). In explaining why these terms are unacceptable in a healthcare context, Schnabel et al. (2021) claim that they may be considered inappropriate because their use might imply a more intimate relationship than exists, or reinforce the differential power dynamic between the patient and healthcare professional (HCP) that comes with the institutional setting.

Studies of usage of ToE in a non-healthcare context report different findings. For instance, they have been shown as a friendly way to demonstrate or build closeness (Febrianti and Auwal, 2021; Khalil and Larina, 2022); to express respect in Syrian-Arabic (Khalil and Larina, 2022) and show affection in Norwegian goodbyes (Svennevig and Johansen, 2012). Rendle-Short (2010) investigated use of the term ‘mate’, in Australia, finding that it is largely interpreted as positive/friendly, and occurs within many contexts such as openings, closings, assessments, agreements and the mitigation of requests and disagreements. Notably, the use of mate was sequentially dependant, and this could influence its interpretation as positive or negative. Overall, this non-healthcare literature suggests that perceptions of ToE are both culturally and contextually dependant. Therefore, interaction must be studied within its real-world context to see when ToE are used, and how they are received by specific populations.

Brown and Draper (2003) conducted a review based on speech-accommodation theory (Ryan et al., 1995), which focussed on use of patronising language towards older adults. They claimed that older adults typically dislike ToE, giving them negative evaluations (e.g. irritating/patronising). However, most evidence involved studies providing participants with hypothetical conversation examples to be rated. This neglects to consider the impact of context in an ongoing interaction. PLWD in hospital, potentially experiencing disorientation, confusion or pain, may receive ToEs differently. Brown and Draper (2003) claim that the use of ToE functions as a way of controlling patients by staff adopting a parent-like role (Brown and Draper, 2003). However, this claim appears to be mostly based on a single article (Kenwright, 1998).

Conversely, if care is viewed as a collaborative activity (Bury and Elston, 1997) then this notion of control discussed by Brown and Draper (2003) could instead be considered cooperation. Evidence for whether PLWD respond to ToE as controlling or collaborative is limited, although one piece of observational research by Carpiac-Claver and Levy-Storms (2007) found that endearments used by staff in USA nursing homes occurred particularly with task-oriented directions, and some residents responded positively. In situations other than healthcare, asking someone to do something could be viewed as a co-operative rather than controlling endeavour. For instance, requesting a favour from a friend (e.g. Harissi, 2005), or requesting assistance from a work colleague (e.g. Risberg and Lymer, 2020). Much of the research into requests incorporates Brown and Levinson’s (1987) politeness theory, which sets out how actions (including requests) threaten the ‘face needs’ (as originally defined by Goffman, 1955) of participants in an interaction. Brown and Levinson also argue that within a given interaction, participants usually cooperate to mutually maintain face, including the mitigation of speech acts which could threaten face. For instance, producing requests with low entitlement so that they are easier to decline.

Harris (2003) uses politeness theory as a starting point to examine requests within various institutional settings involving power imbalances, noting how institutional members (including doctors) used strategies (including mitigation) to offer clients a means of redress during face threatening acts. The concept of mitigation in conversation is longstanding. Fraser (1980: 341) wrote: ‘*Mitigation is defined not as a particular type of speech act but the modification of a speech act: the reduction of certain unwelcome effects which a speech act has on the hearer*’. Caffi (1999) claims that mitigation is a synonym for attenuation, and results from ‘*a weakening of one of the interactional parameters’* (p. 882). Writing more recently, Estellés and Albelda (2022) note that mitigation can be done through many mechanisms. For example, modifiers, quantifiers, modal verbs, adverbs or prosodic devices (e.g. lowering the voice). However, and significantly, they state that mitigation does not depend on any specific mechanism. Instead, it is dependent on interactional context, and is done in a way that appears intentional and strategic to (1) reduce an aspect of communication, or (2) achieve an interactional goal or (3) protect the image/face of participants.

### Methodology

Data were collected as part of the NIHR funded VOICE (13/114/93; Harwood et al., 2018; O’Brien et al., 2018) and VOICE2 (NIHR134221) research projects (See Allwood et al., 2017; O’Brien et al., 2018, 2020; Pilnick et al., 2021). Ethical approval for this analysis was provided by the NHS Yorkshire and Humber–Bradford Leeds and the Wales7 research ethics committees (REC) and the University of Nottingham School of Sociology & Social Policy REC.

Patients were recruited if they had a diagnosis of dementia mentioned in their medical notes and were identified by hospital staff as having a level of communication difficulty (VOICE) or as experiencing distress whilst in hospital (VOICE2). All patients were over the age of 65. In each case, as part of the recruitment process, the mental capacity of patients was assessed by a member of the research team. If they were deemed lacking in capacity to consent to the study, then consultee agreement was sought (as per section 32, Mental Capacity Act, 2005). All patients apart from one in study 2 lacked the mental capacity to give informed consent to participate in the study. Patients were not recruited if they were judged to be at end of life by the medical team, or they did not speak English in interactions with HCPs. A variety of HCPs were recruited on the wards, including doctors, nurses, healthcare assistants (HCAs), speech and language therapists, occupational therapists and physiotherapists. Of these, all were recorded using ToE, aside from occupational and physiotherapists. A total of 85 recordings were available for this analysis (with study 1 and study 2 combined). This involved 77 HCPs and 43 patients, and included approximately 14 hours and 46 minutes of video recording. No participant was videoed more than three times.

All interactions presented here took place on healthcare of the older persons wards in two UK hospitals. They all involved talk between PLWD (patients) and healthcare staff working on the wards. Some interactions involved multiple HCPs working with a patient. In each interaction, HCPs were working with the patients to complete various daily healthcare tasks such as medical examinations, patient assessments, personal care and assisting the patient with eating/drinking. Intimate care was not filmed in study 1 and was recorded as audio only in study 2 to preserve patients’ privacy and dignity.

In the data set, 29 out of 85 video/audio recorded interactions included the use of ToE from a HCP. Terms used included ‘darling’, ‘sweetheart’, ‘my lovely’, ‘love’, ‘lovey’, ‘duck’, ‘mate’, ‘young lady’, ‘good man’ and ‘my dear’. These 29 interactions were examined using conversation analysis (CA). This method involves detailed consideration of the orderly, structured nature of talk (Stivers and Sidnell, 2013). Both video/audio data and transcripts (transcribed according to Jefferson’s (2004 conventions) were examined together, according to Sidnell and Stivers’s (2013) three stages (observation of the data, identification of interesting phenomena and collection of examples). These examples then allowed the description of practices through analysis of singular examples and comparison across multiple cases. The preliminary analysis was presented at data sessions involving members of the wider research team and other researchers working within this area, allowing further refinement and development of shared understanding (Ten Have, 2007). The benefits of using CA within this type of research setting are illustrated by Drew et al. (2001), but in short, CA allows the exploration of how HCPs use particular turn designs in different situations, and the sequential implications of these designs (i.e. how patients react). This is done by examining directly observable properties of the data, and so does not involve potentially subjective interpretations of participants’ internal states. Atkins (2019) further demonstrates how CA can be used to better understand what actions a given conversational phenomenon performs.

## Analysis

This paper describes 10 examples in which ToE have been identified as a mitigation device. It has been shown that ToE can serve a mitigating function in other (potentially face threatening) contexts (McCarthy and O’Keeffe, 2003; Rendle-Short, 2010). The use of ToE within these data reflects this, as ToEs occurred during the context of requests to repeat talk, and during healthcare activities in which the HCP was giving an instruction or producing other talk relating to task completion.

### When asking a patient to repeat

Multiple examples of HCPs using a ToE when asking a patient to repeat a turn they had misheard or misunderstood were identified: two examples are shown below. This phenomenon has also been found in other circumstances. Baumgarten (2021) identified situations in which individuals used the endearment ‘love’ during clarification requests. However, within Baumgarten’s data these requests only occurred within non-institutional settings. She also noted that they were usually a result of mishearing: lack of knowledge or inattentiveness from the older party in parent-child or older adult-younger adult groups. Although her focus was specifically the term ‘love’, this raises questions around the use of ToE in the present data, which were collected in an institutionalised healthcare environment. One possibility is that it is the local interactional context, rather than the wider hospital one which is important.

In Extract 1 below, the HCP (a male doctor) is checking the female patient’s breathing and has a stethoscope in his ears. He takes it out and asks the patient to repeat her utterance in line 162.


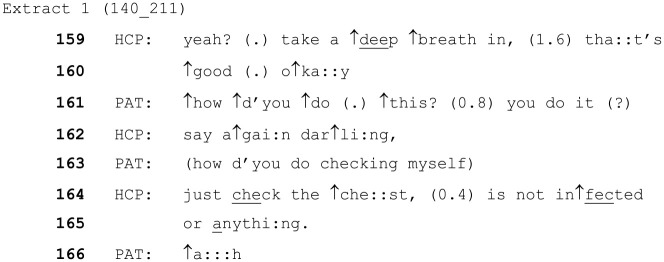


The patient’s talk on line 161 is hard to interpret (Pilnick et al., 2021) and the HCP asks her to repeat with the ‘say again darling’,. The patient responds with something also difficult to interpret on line 163, but the HCP then chooses to respond to the word checking, framing a response that echoes this word in explaining his actions. The fact that he does this is notable, since picking up on an aspect of hard to interpret talk and repeating it back has been shown in previous research (Pilnick et al., 2021) to be a way of maintaining patient’s ‘face’ (Goffman, 1955).

A similar situation occurs below. In the following extract, the HCP (a female speech and language therapist) is attempting to assess a (male) patient’s swallowing by giving the patient some water.


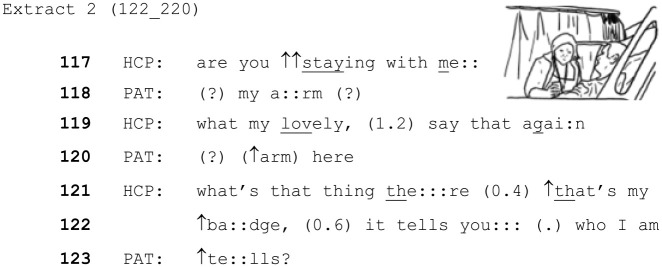


The HCP asks the patient if he is ‘staying with her’, since he appears quite sleepy. The patient then says something that is unclear, and the HCP uses the endearment ‘my lovely’ when she asks him to repeat. When the patient’s response is again difficult to interpret, then, as in the example above, she also attempts a response based on what she has understood from the patient’s talk.

Past research has demonstrated that managing repair with PLWD is difficult (e.g. Perkins et al., 1998; Schrauf, 2020), particularly within this hospital context (Pilnick et al., 2021). In these situations, and in line with previous literature on mitigation (e.g. Estellés and Albelda, 2022) the endearment works towards mitigating the repairs used by HCPs by reducing the impact of these statements. Repair is potentially face-threatening (Goffman, 1955) because it draws attention to a lack of shared understanding, and this can imply problems with the speaker, rather than the hearer. It is also notable that in both above examples, following the first repeat request the HCP then attempts an answer based on what they have been able to understand, rather than making further clarification requests which could draw further attention to the difficulty and more clearly locate the problem with the patient’s talk. This aligns with the research linking mitigation with saving face described above (e.g. Brown and Levinson, 1987; Harris, 2003; McCarthy and O’Keeffe, 2003) and also avoids the need for further repair (Pilnick et al., 2021).

### Instructions during healthcare tasks

The use of ToE during healthcare-based instructions, requests and general task-based talk were common in these data. Examples of healthcare-based instructions will be examined first, followed by other task-based talk.

The following extract is from the same interaction as extract 2 above. The HCP has already attempted to offer the patient water multiple times (to assess his swallowing), but the patient has refused. At this point, the HCP is returning to offering the water after trying other foods.


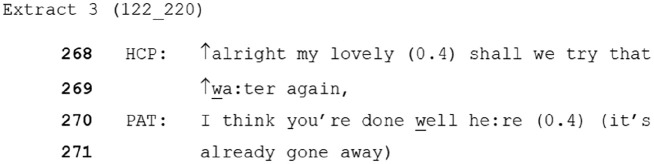


The HCP uses the endearment ‘my lovely’ as a part of her proposal to try the water again. Additionally, the HCP uses the collective ‘we’, as part of her request but the ‘we’ in this instance denotes a collaborative action. The HCP needs to hold the glass to assist this patient. Bowie (1996) argues that HCPs (in their case, nurses) choose forms of address that are higher in solidarity when they wish to impart commonality of purpose and a sense of closeness with patients. Hence, it seems that the request here is designed to encourage cooperation between the HCP and the patient. Nonetheless, Bowie suggests that some (particularly older) patients, can find this type of talk an infringement of propriety or potentially patronising.

With this in mind, the question could be raised here about control. Brown and Draper (2003) claim that ToE can be used due to an underlying ageist attitude and aim to exert control over older people. However, many of the tasks carried out in the current dataset were considered medically necessary, and required a certain amount of collaboration between HCP’s and patients for them to be completed safely. In this context ToE may function to create a favourable environment for cooperation, rather than being an agent of direct control. There may be no way of avoiding giving a patient an instruction, but the addition of a ToE can soften this.

Giving instructions has important implications for the asymmetry of an interaction. Being cared for necessarily results in some relinquishing of agency and control on the part of the person receiving the care (Antaki and Webb, 2019). The carer (in this case the HCP) also likely has greater knowledge of the care activity that needs to be completed or the rationale for it. However, if the patient is unsure or unwilling, then directly instructing them is face threatening because of its impact on individual agency (Armstrong, 2014; Landmark et al., 2015). Mitigating features of talk in this situation serve to reduce this threat to agency and face, by softening instruction sequences that could otherwise sound harsh or inappropriate. Another example of this can be seen below.

In this example, the male patient (PN03) is lying in bed with a female nurse (HN20) on his left and a female healthcare assistant (HN18) on his right. Their goal is to move the patient and help him into a sitting position so they can then assist him with his dinner.


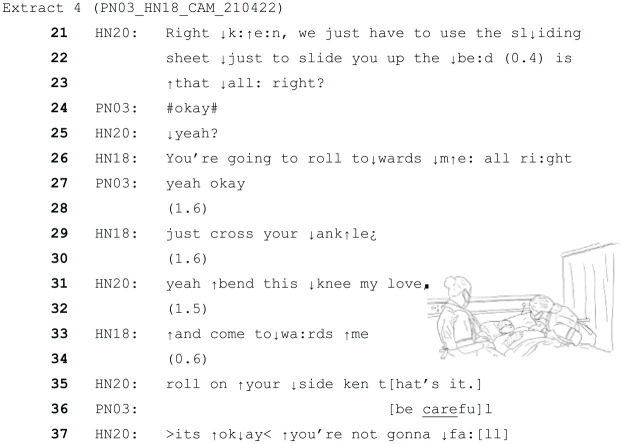


In extract 4 above, both HCPs work together to turn the patient onto his side safely. HN20 uses the word ‘just’, (line 22) minimising the suggested task (see O’Brien et al., 2020), and both seek confirmation from the patient before beginning (‘all right?’ lines 23 and 26), which the patient responds to with agreements (lines 24 and 27). HN18 also uses the minimiser ‘just’ (line 29) when asking the patient to cross his ankles, thereby downplaying the effort that must be taken. HN20 produces her utterance in line 31 as a continuation of the ongoing sequence of instructions, but uses the ‘my love’ endearment at the end of hers, also having a mitigating effect.

These mitigators are significant here, because as the HCPs give the instructions, they then also do the task with the patient contemporaneously. He is therefore not really in control of his own movements; the HCPs are moving his body to complete the task. Having one’s body moved in this way is potentially highly threatening to patient agency. However, the talk is framed as if the patient is working with them to complete the task. For instance, when HN20 says ‘bend this knee my love’, she is bending his knee, but by framing it as a mitigated instruction, it constructs it as a collaborative action in which the patient’s agency is exercised by co-operation.

Extract 5 below is similar to extract 4 above as it involves two female healthcare assistants (HN63 and HN64) working together to move a (female) patient (PN13) around on a bed. However, this case provides an example of care where the patient is actively resisting. For context, the HCPs involved here reported afterwards that the patient’s distress began when they had to turn her onto her ‘bad’, (more painful) side. Most of this interaction was recorded as audio only due to personal care. However, prior to this extract, the HCPs have discussed between themselves how the patient is a ‘fighter’ suggesting ongoing difficulty is expected. The patient’s turns reinforce this perception, as she accuses the HCP’s of hurting her and uses multiple swear words. This recording is audio only.


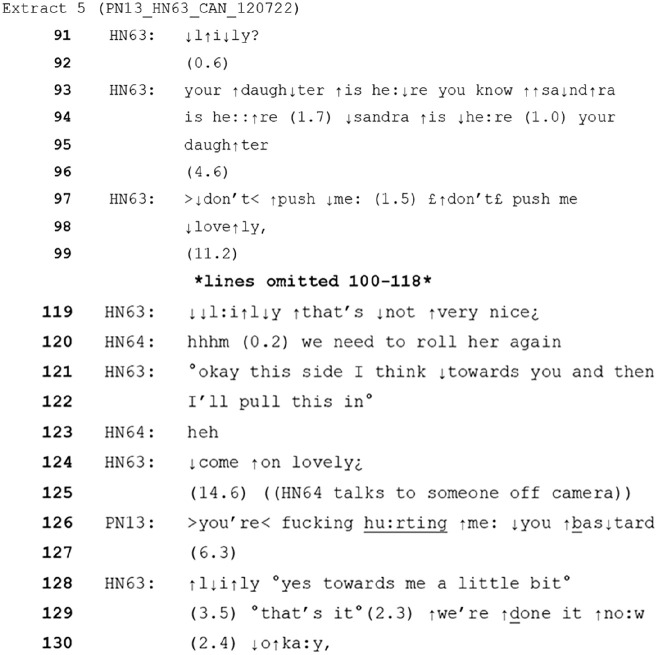


When the extract starts, HN63 is telling the patient her daughter is visiting whilst she attempts to move or change her. After a pause HN63 responds to a physical action from the patient by saying ‘don’t push me’, which is then repeated in a softer voice with the endearment (lovely) added. There is a second ToE on line 124 (‘lovely’).

It is notable that this is likely a difficult situation for all involved. The patient demonstrates mid-task that she has become distressed, with physical resistance and verbal aggression. Despite this, the HCP’s have begun the process of changing her, and arguably could not stop mid-task as this would leave the patient still uncomfortable and exposed, threatening her safety and dignity. They therefore must somehow complete the task, and account for the fact that the patient is in continued distress and is resisting their actions.

The incorporation and positioning of these ToEs works to soften and reduce the severity of the healthcare assistant’s instruction to not push. In other contexts, physical actions such as pushing could be seen as aggressive, unsettling or threatening (e.g. Zuzelo et al., 2012) and a more direct or confrontational response would be expected. Additionally, in this context, the HCPs are continuing an activity that they know will potentially cause discomfort. Using a ToE here is a means of acknowledging this by implying a caring relationship or solidarity (see Bowie, 1996) towards the patient. Evidence for this can be seen in their characterisation of the patient’s turns as inappropriate, where the inappropriateness is minimised. For instance, healthcare assistant HN63 says ‘that’s not very nice’ (line 119) in response to the patient swearing. In addition, their continued lack of a reciprocal negative response is notable. Whilst patient distress here is not avoided, it does not escalate further, and the task of providing the patient with clean clothes and bedding is completed successfully.

### Responses to patient unease during healthcare tasks

The following analysis will consider situations where the HCP was trying to achieve a particular medical task or goal but was not giving an explicit instruction or request to the patient. In extract 6 below, the (female) patient is sitting in a chair at the side of her bed and is having a blood sugar test.


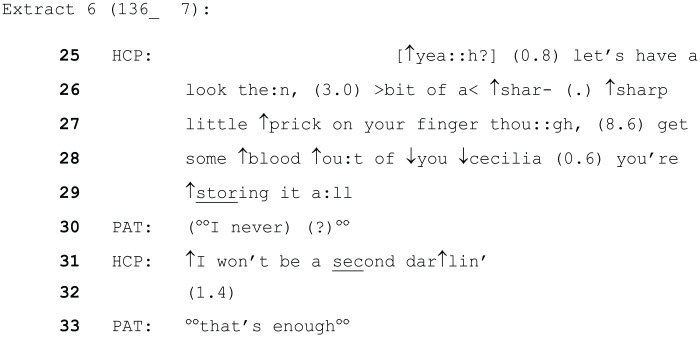


The HCP (a female nurse) uses a different form of address initially (the patient’s first name, anonymised as Cecilia here). This occurs when the HCP is narrating her actions, whilst she tries to get the required amount of blood from the patient’s finger. The ToE (darling, line 31) occurs just after the patient produces some unclear talk and is looking around touching the nearby curtain as if attempting to begin an action. It seems likely that the HCP’s turn is responsive to this initiation and is working to delay the patient from this whilst simultaneously implying the soon-to-be-completed nature of the blood collecting task as she finishes it. The patient responds by returning to attending to the task, and after another short pause observes ‘that’s enough’, when some blood has been collected.

Again, this ToE is working in conjunction with the rest of the language in this extract to foster an environment of co-operation between the patient and HCP. The language is again minimising, for instance when the HCP says, ‘bit of a sharp little prick’ (lines 26 and 27). Moreover, the phrase ‘I won’t be a second’, emphasises the small or brief nature of the task. It is therefore likely that it orients to the fact that something painful but necessary is occurring, which the patient wishes to stop but cannot. The endearment ‘darling’, works to mitigate the fact that the HCP is overriding the initiation attempt made by the patient. This again relates to patient agency. The HCP’s use of ‘I’ (line 31) demonstrates the activity is something she is doing (as opposed to a joint action with the patient), which carries the implication that the HCP’s activity is of more importance or significance in that present moment than whatever the patient was going to say. Although this may be understandable logistically (the HCP could not pause in the middle of blood collecting), the result is a lack of agency for the patient in that instant. The mitigating ToE may therefore serve as a means of recognition and redress (Harris, 2003) in this context.

Furthermore, although the HCP’s statement (including the ToE) has overridden the patient’s potential initiation, it is politely delivered (Brown and Levinson, 1987), in the sense that it attends to the need to wait whilst the task is completed whilst minimising the imposition on the patient.

This skill of resolving potentially conflicting needs or actions and maintaining a positive co-operative interaction is of huge importance within this environment, where the particular communication and cognitive difficulties of patients have already been discussed and demonstrated. Further examples of this kind of reassurance were seen in cases where the HCP appears to be aiming to mitigate the patient’s distress around a particular issue. In the following example, the male patient has become convinced that he is going to have to take over the job of Prime Minister of the UK and is quite distressed by this idea as he states he is unable to meet the demands of the role. The HCP (a female HCA) working with him has been trying to convince him that he won’t have to do the job.


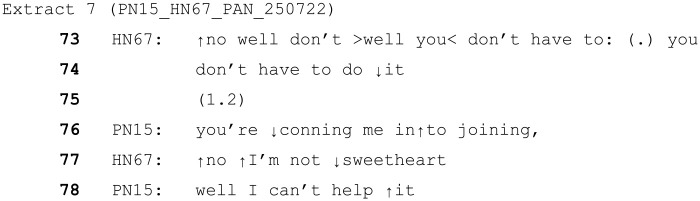


In this case, the ToE (‘sweetheart’, line 77) is used as part of the HCP’s disagreement with the idea that she’s trying to get him to join the government. The use of ‘sweetheart’ softens her objection to his assumption, mitigating any conflict that could arise due to her disagreement. Again, her turn in this case directly deals with his concern. The patient does not pursue the idea that the HCP is conning him, and instead continues sharing his worries regarding what would happen if he declined to take the job.

A similar sort of need for reassurance occurs in the following interaction, in which a female nurse has just changed the continence pad of a male patient. The patient raises concerns that he has done something wrong (i.e. he has soiled himself). The nurse reassures him that he is clean now (not shown here) and he hasn’t done anything wrong. This recording is audio only.


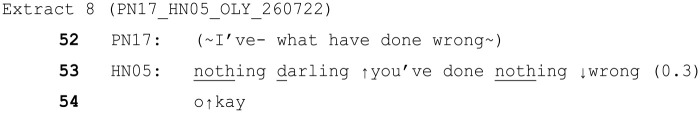


The ToE again occurs when the HCP disagrees with an assumption or statement made by the patient. Similarly to the extract above, her words are contradicting the patient’s turn but the use of the ToE (‘darling’) softens this. Although it is in opposition to what the patient has said, the HCP’s statement is reassuring, as it implies that since the patient has done nothing wrong, there is no problem with the situation.

It therefore appears that overall, when used in response to patient unease, ToEs are a way of potentially avoiding (extract 6) or managing (extracts 7 and 8) conflict. In the case of extract 6, the ToE attends to the fact that the HCP is overriding the patient’s cues or wishes. Extracts 7 and 8 demonstrate ToEs softening apparently contradictory statements as in both cases, the HCP’s response directly targets the patient’s concern. It should be noted however, that that this ideal of directly answering a patient’s concern may often be difficult to meet in this context.

### When ToE are rejected

The following section provides two examples in which ToE are treated as problematic by patients. In the following extract, the woman living with dementia (PN05) is having a (medically required) cannula inserted. At this point in the interaction, two mental health nurses (HN12 (male, left) and HN24 (female, right)) are talking to her whilst restraining her by each holding one of her hands whilst a doctor (not in transcript) inserts the cannula. The patient has protested repeatedly. It should be noted that (as with many other patients in this data) this patient did not have the mental capacity to decide on the treatment needed. The hospital staff had a deprivation of liberty safeguard (DoLS) authorisation in place (MCA, 2005 as amended by MHA 2007) for them to give her this medical treatment in her best interests.


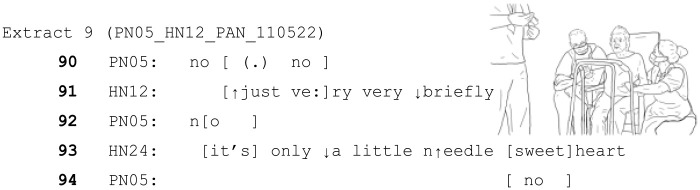


In this case, both HCPs are using minimising language, suggesting the short time frame and small nature of the task they are trying to complete. (e.g. “only a little needle sweetheart). However, the patient protests (and continues to do so throughout the interaction).


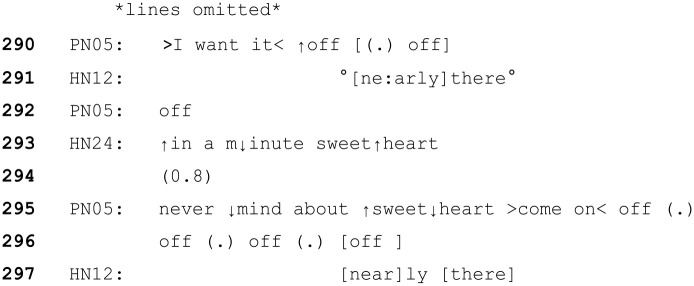


Once the cannula is inserted, the patient repeatedly states she wants it off. The HCPs continue to take turns which are designed to delay compliance with the request to remove the cannula or let her go (e.g. ‘in a minute sweetheart’), and emphasise the minimal, soon-to-be-completed nature of the task (e.g. ‘very briefly’, ‘nearly there’.). Nevertheless, unlike other examples where this indication of brief temporal delay results in a patient cooperating/not objecting, in this case the patient does object, and notably specifically identifies the ToE ‘sweetheart’, as inappropriate (line 295). Her dismissal of the word ‘sweetheart’, followed by ‘come on off, off. . .’ suggests she has treated the ToE as irrelevant to the business at hand (her objective to get them to remove the cannula) and has rejected the HCP’s attempts to delay her from pursuing her objective.

The fact that the ToE is explicitly treated as inappropriate in this situation, but not others, is worth consideration. It is possible that this rejection was due to the specific nature of the task, or the individuals involved. It could be argued that this is a particularly invasive task that is less routine or familiar than others that might form a regular part of the patient’s care. The patient’s objections are also strongly, clearly and repeatedly expressed, so the fact that the task continues in the face of these (albeit in her best interest medically) means that there is an obvious and strong threat to her sense of agency. However, as in Extracts 5 and 6 above, this is a task that cannot easily be stopped once it has been started.

When a patient has capacity to decide their medical treatment, HCPs would not administer treatment against their will, and to do so would be considered an assault on the patient. The exceptions are individuals who lack the mental capacity to decide for themselves and children under the authority of their parents. As previous literature has established, talking to older adults in a way that positions them as a child (i.e. elderspeak) does have possible negative connotations such as diminishing self-esteem, belittling or othering (Ryan et al., 1995) and threat to their sense of personhood (Williams et al., 2016).

Both of HN24’s turns involving ToE ‘it’s only a little needle sweetheart’ and, ‘in a minute sweetheart’, do not attend to PN05’s immediate demands (that she does not want the cannula, and she wants it off). This (along with their continued actions) demonstrate to PN05 that the HCP’s are not following her stated wishes. It is therefore feasible that the patient’s reaction to ‘sweetheart’ is related to this clear rejection of her assumed agency as an adult to refuse medical treatment. In this case, the mitigators (including ‘sweetheart’,) were not enough for the patient to orient to the HCP’s actions as acceptable. In short, ToEs will not always achieve their intended aim.

The following is another example of a situation in which a ToE is involved where the patient is objecting to treatment (in this case an injection) and where the HCP attempts to persuade them. As above, the patient objects to the HCP’s turn, treating it as unsatisfactory or potentially inappropriate, though in this instance the ToE itself is not explicitly problematised.


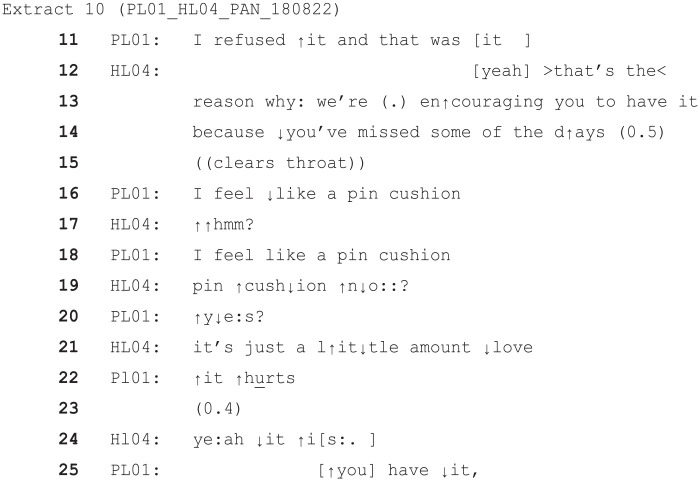


In this case, the (female) patient (PL01) does have more initial agency, since this is a discussion about a potential treatment before the treatment goes ahead. She demonstrates this by outlining some reasons to refuse the injection, namely she has refused it successfully in the past, and (when countered by HLO4) she feels like a ‘pin cushion’. HL04 (a female nurse) replies with a mitigating response downgrading the description of the procedure and involving the ToE (‘love’) (line 21). The patient objects to this turn quite strongly with ‘it hurts’ in a raised voice, and when HL04 tries to respond to this she adds the further ‘you have it’, implying she has not taken HL04′s mitigations as adequate. This rejection of the HCP’s mitigated attempts appears to be grounded in the patient’s view that her objections have not been adequately addressed. Adults with capacity to make their own medical decisions would be free to reject care they deemed unwanted or unnecessary, and these examples show that ToEs will not necessarily work to mitigate what are deemed to be inadequate accounts for the delivery of care where patient requests are overridden.

## Discussion

Overall, ToE were found serving a mitigating function in a number of contexts. This included situations in which conversational repair was needed, for example in response to hard-to-interpret talk (Pilnick et al., 2021) on the part of PLWD. It also included contexts in which HCPs were attempting to complete a healthcare task with a PLWD, particularly where this task represented a challenge to the agency and control of the PLWD, such as when patients were instructed to do something or were having something uncomfortable done to them. However, as the analysis has shown, the use of ToEs does not always result in successful mitigation. Mitigation is sensitive to the specific interactional circumstances of the interaction, and potentially the responsiveness of the HCP to the patient’s voiced concerns.

If mitigation is successful, then it is a potential aid towards fostering an environment of cooperation between PLWD and HCP’s. Although it cannot remove the issue of an individual’s agency being overridden, it can acknowledge and respond to this and may reduce the level of threat to face (Goffman, 1955). This conclusion is highly relevant with respect to previous literature framing ToE as patronising, inappropriate (Schnabel et al., 2021; Shaw et al., 2022; Williams et al., 2016, 2017a) and controlling (Brown and Draper, 2003). What has been shown here is that, if used successfully, ToE can support patients by acknowledging the sensitivity of situations in which expressed wishes are overridden, or an objected to course of action is continued. However, patients may not accept this mitigation, so ToEs are not a ‘magic bullet’ to avoid treatment refusals and distress.

The underlying assumption of much healthcare research (e.g. Bury and Elston, 1997; Landmark et al., 2015) is that patients have a (potentially educated) awareness of the situation, and a knowledge of whatever condition is troubling them which is complete enough to allow debate over decisions such as treatment plans (e.g. Stevenson et al., 2021). This is unlikely to be the case for PLWD in this study, who did not always demonstrate insight that they were in hospital receiving medical treatment. This resulted in various challenges such as patients threatening to phone the police or becoming otherwise distressed when they were unable to leave the ward, or not recognising medical issues (e.g. a patient claiming there was nothing wrong with her arm which was in a plaster cast). Where patients lack capacity to make decisions about medical treatments, decisions will be made in their best interest which may conflict with a patient’s expressed wants. These are difficult situations for HCPs to manage successfully, but mitigation through using ToE is one way in which they attempt this. This context-sensitive deployment of ToEs underlines the high quality of interactional skill professionals demonstrate whilst working within this area, as they respond to the contingencies of interaction with individual patients.

The question of whether the findings from these data can be applied more widely is relevant here. The wider generalisability of these findings to other inpatient dementia care settings is likely to be limited by the lack of diversity within the patient group, who were all White and largely spoke English as a first language. It seems likely, for example, that there may be cultural variations in the use and acceptance of ToEs.

Nonetheless, the finding that ToEs can serve important interactional functions is likely to be more widely applicable in care contexts. For example, in other non-hospital care environments involving PLWD, the need for repair or the issue of agency will also become relevant. Future research should aim to examine whether ToE are used in a similar way in these environments. The present findings are also relevant for wider healthcare settings, where patients are placed in a vulnerable position through pain or illness, and may require care outside of their control, making mitigation relevant. Additionally, further research could examine interactions with or without ToE in terms of broader outcomes, as this paper focussed on the local interactional context only, which is a limitation.

Even so, with the strong evidence of negative experiences of PLWD in hospital (e.g. Dewing and Dijk, 2016; Featherstone and Northcott, 2020; Røsvik and Rokstad, 2020; Sampson et al., 2009), an additional tool which could aid in communication with PLWD in difficult contexts is likely be useful for HCPs. It is also important that any prohibition or discouragement of interactional practices, such as the idea that ToEs are always inappropriate, should be grounded in empirical evidence.

To conclude, administering healthcare to PLWD in the acute hospital environment can be challenging for staff, and a range of approaches are likely to be needed to respond to individual contextual circumstances. However, the interactional evidence presented here does not support a blanket ban on ToE, since they serve an important purpose as a feature of mitigation in the contexts presented here.
